# Wellbeing as antidote against burnout: interventions and actions

**DOI:** 10.1192/j.eurpsy.2025.261

**Published:** 2025-08-26

**Authors:** L. Cuffaro

**Affiliations:** 1 Neurology Department & Brain Health Service, University of Milan Bicocca, Monza; 2 Candido Neuro Care Institute, Agrigento, Italy

## Abstract

**Abstract: Background:**

Burnout among mental health professionals poses a significant threat to personal wellbeing, patient care, and healthcare systems. Addressing this challenge aligns with the broader mission of the European Academy of Neurology (EAN) to promote brain health not only for patients but also for clinicians. This presentation highlights education, collaboration, and systemic interventions, integrating neurological and psychiatric perspectives.

**Methods:**

This initiative stems from the EAN Task Force for Wellbeing (TFW), in collaboration with the European Psychiatric Association (EPA). A multipronged strategy was designed to enhance awareness, build resilience, and foster systemic reforms:

1. Educational Media: A podcast series exploring burnout’s conceptualization, its neurobiological and psychological aspects, gender disparities, and practical solutions.
2. Interactive Webinars: Interdisciplinary sessions combining insights from neurology and psychiatry, showcasing evidence-based strategies and technological innovations.
3. Public Debates: Open forums to engage professionals across career stages, raising awareness and encouraging action to tackle burnout.

These initiatives were supplemented by systematic reviews, case studies, and suggestions of novel tools to monitor mental health and stress factors.

**Results:**

The combined educational and intervention efforts yielded significant outcomes:

1. Advancing Brain Health for Clinicians: Awareness initiatives emphasized the vital link between clinician brain health and professional resilience
2. Skill Development and Resilience: Practical strategies for time management, mentorship, and self-care were disseminated through case studies and interactive discussions.
3. Collaborative Solutions: Webinars and public debates highlighted shared challenges and fostered interdisciplinary advocacy for systemic reform.

**Discussion:**

Burnout is not just an individual issue but a systemic challenge requiring collective action. By prioritizing clinician brain health as a mission, the EAN and EPA collaboration provides a unifying framework to tackle burnout. Educational initiatives bridged the gap between theory and practice, while public debates fostered community engagement and systemic advocacy. Interdisciplinary collaboration underscored the shared nature of burnout and the need for unified, brain-health-centric strategies.

**Conclusion:**

Promoting wellbeing through education, collaboration, and innovative tools aligns with the broader goal of achieving brain health for clinicians. These initiatives provide a sustainable pathway to counteract burnout and foster a resilient and thriving workforce. Continued investment in education, public discourse, and systemic reform is essential to realizing this mission.

**Keywords:**

burnout, brain health, wellbeing, education, interdisciplinary collaboration, neurology, psychiatry, systemic reforms

**Disclosure of Interest:**

None Declared

